# Improving acid resistance of *Escherichia coli* base on the CfaS-mediated membrane engineering strategy derived from extreme acidophile

**DOI:** 10.3389/fbioe.2023.1158931

**Published:** 2023-03-21

**Authors:** Wenbo Hu, Yanjun Tong, Junjie Liu, Panyan Chen, Hailin Yang, Shoushuai Feng

**Affiliations:** ^1^ The Key Laboratory of Industrial Biotechnology, Ministry of Education, School of Biotechnology, Jiangnan University, Wuxi, China; ^2^ State Key Laboratory of Food Science and Technology, Jiangnan University, Wuxi, China; ^3^ Key Laboratory of Carbohydrate Chemistry and Biotechnology (Jiangnan University) Ministry of Education, Jiangnan University, Wuxi, China

**Keywords:** CFAs, membrane engineering, CRISPR-Cas9, acid stress, extreme acidophile

## Abstract

Industrial microorganisms used for the production of organic acids often face challenges such as inhibited cell growth and reduced production efficiency due to the accumulation of acidic metabolites. One promising way for improving the acid resistance of microbial cells is to reconstruct their membranes. Herein, the overexpression of *cfa*2 from extreme acidophile endowed *E. coli* with high-performance on resistance to the acid stress. The engineered strain M1-93-*Accfa*2, constructed by CRISPR/Cas9-mediated chromosome integration, also exhibited a significantly higher resistance to severe acid stress. The analysis of fatty acid profiles indicated that the proportion of Cy-19:0 in the cell membrane of M1-93-*Accfa*2 increased by 5.26 times compared with the control, while the proportion of C18:1w9c decreased by 5.81 times. Correspondingly, the permeability and fluidity of the membrane decreased significantly. HPLC analysis demonstrated that the contents of intracellular glutamic acid, arginine, methionine and aspartic acid of M1-93-*Accfa*2 were 2.59, 2.04, 22.07 and 2.65 times that of the control after environmental acidification, respectively. Meanwhile, transmission electron microscopy observation indicated that M1-93-*Accfa*2 could maintain a plumper cell morphology after acid stimulation. M1-93-*Accfa*2 also exhibited higher-performance on the resistance to organic acids, especially succinic acid stress. These results together demonstrated the great potential of M1-93-*Accfa*2 constructed here in the production of organic acids.

## Introduction

In the fermentation industry, model microorganisms such as *E. coli* are frequent engineered to produce organic acids, which is generally considered a promising biorenewable alternative to current petroleum-based methods (Gallezot, 2007). However, with the accumulation of acidic metabolites, the pH of the fermentation broth decreases, which will eventually inhibit cell growth and limit the performance of the production strains (Yan et al., 2016). Various approaches have been developed to address this challenge, including adding alkaline reagents such as NaOH, Na_2_CO_3_ and CaCO_3_ to neutralize the acidic environment or through membrane engineering (Tan et al., 2016), global transcription machinery engineering (Wu et al., 2020), adaptive evolution (Hua et al., 2006), chemical mutagenesis (Klein-Marcuschamer and Stephanopoulos, 2008), and the addition of exogenous auxiliary energy substrates (Sánchez et al., 2008) to reinforce the acid resistance of production strains (Yang et al., 2021).

Although the toxicity of products can cause multiple adverse effects on the production strains, membrane damage is generally considered to be the general mechanism of toxicity due to its outermost localization (Tan et al., 2016). As a key barrier to the homeostasis of the intracellular environment, cell membrane plays a crucial role in signaling, material transport and energy exchange (Zhu et al., 2011; Hu et al., 2020; Huo et al., 2022). Therefore, cell membrane has become a popular engineering target to improve the resistance of production strains to the acidic metabolites (Sandoval and Papoutsakis, 2016; Santoscoy and Jarboe, 2021). Previous studies have mostly focused on modifying microbial cell membranes by altering the chain length of membrane components to increase membrane integrity and mitigate leakage (Sherkhanov et al., 2014; Royce et al., 2015). However, the change of membrane lipid composition in other routes could also have a significant effect on the properties of the membrane. For example, higher levels of cyclopropane fatty acids (CFAs) in cell membranes can enhance the compactness of the membranes and form an effective defensive barrier against H^+^ that is widely distributed in the environment (Shabala and Ross, 2008; Arcari et al., 2020). Cyclopropanation is catalyzed by cyclopropane fatty acid synthase (CfaS). Specifically, the enzyme adds a methylene group derived from S-adenosylmethionine (AdoMet) to the double bond of an unsaturated fatty acid (UFA) incorporated in a phospholipid to form a CFA (Law, 1971; Grogan and Cronan, 1997; Bianco et al., 2019) (Supplementary Figure S1). If this CfaS-mediated cell membrane remodeling strategy is applied to the membrane engineering of organic acid-producing strains, the resulting improved microbial acid resistance will not only help to save processing costs by reducing the alkali usage, and alleviate or avoid osmotic stress associated with the use of alkaline reagents, but also decrease the vulnerability to contamination.

Extremophiles are able to thrive in harsh environments where other organisms cannot even survive, these microbiotic-derived enzymes convey the characteristics of surviving in extreme environmental conditions and have the potential to be a valuable resource for the development of a bio-based economy (Raddadi et al., 2015; Sysoev et al., 2021). For example, the expression of heat shock proteins (HSPs) or redox proteins from *Thermoanaerobacter tengcongensis* in *Saccharomyces cerevisiae* has shown excellent effects on improving thermo-tolerance (Xu K. et al., 2020). Another study showed that heteroexpression of *cfa* from *Halomonas socia* in *E. coli* enhanced its tolerance to inhibitors such as furfural, 4-hydroxybenzaldehyde, vanillin, and acetate, and increased polyhydroxyalkanoate (PHA) production by 1.5 times (Choi T-R. et al., 2020). In addition, our previous transcriptomic study of *Acidithiobacillus caldus*, an extreme acidophile, in response to acid stress had shown that its *cfa* gene expression was up-regulated with the intensity of acid stress (Feng et al., 2021).

In this study, we cloned the CfaS-encoding gene from the genome of *A. caldus* CCTCC AB 2019256, and constructed a variety of engineered strains with significantly improved acid resistance by means of plasmid overexpression and chromosome integration. The reasons for the improvement of acid resistance were analyzed from the aspects of fatty acid composition, membrane properties, the contents of intracellular free amino acids and ATP, and cell morphology. Furthermore, we also explored the general effect of this CfaS-based membrane engineering strategy in improving microbial robustness, and the engineered strain M1-93-*Accfa*2 reported here exhibited its potential application value in the production of organic acids, particularly succinic acid.

## Materials and methods

### Bacterial strains, media and growth conditions

All bacterial strains and plasmids used in this study are listed in Supplementary Table S1. *A. caldus* CCTCC AB 2019256 was cultivated at 40 °C and 180 rpm in the modified liquid Starkey-S^0^ medium. The compositions were as follows (g/L): (NH_4_)_2_SO_4_ 0.3, K_2_HPO_4_·3H_2_0 0.5, KH_2_PO_4_ 3.0, MgSO_4_·7H_2_0 0.5, KCl 0.1, Ca(NO_3_)_2_ 0.01, distilled water 1 000 ml. The medium was modulated to pH 1.8 with dilute sulfuric acid and sterilized under steam pressure at 121 °C for 20 min, and 0.5 g S^0^ (sterilized by UV light) was added separately to the 100 ml liquid medium. *E. coli* strains were grown at 37 °C (When the temperature-sensitive plasmids were contained in the bacterial cells, the strains were cultured at 30 °C) and 200 rpm in Luria-Bertani broth (Oxoid) or in E minimal medium with glucose as the only carbon source 0.5% (w/v) (Xu K. et al., 2020). When necessary, antibiotics were added at the following concentrations: kanamycin, 50 μg/ml; spectinomycin, 50 μg/ml. *E. coli* DH5α was used for the vector construction.

### Plasmid construction

The primers used in this study are listed in Supplementary Table S2. The ClonExpress^®^ II One Step Cloning Kit manufactured by Vazyme was used for directional cloning of the target gene. The genes *cfa*1 and *cfa*2 were cloned into the first multiple cloning site (MCS-1) of the pRSFDuet-1 vector using gene-specific primer pairs *cfa*1-F and *cfa*1-R, *cfa*2-F and *cfa*2-R, respectively. The corresponding vector linearization primers were pRSF-F1 and pRSF-R1, and the resulting plasmids were named pRSF-*cfa*1 and pRSF-*cfa*2, respectively. The pTargetF-Δ*ldhA* plasmid was constructed by replacing the N_20_ sequence of gRNA in the original pTarget plasmid with the N_20_ of the target gene *ldhA*. The gRNA used in this study was designed by CHOPCHOP (http://chopchop.cbu.uib.no/). All plasmids constructed herein were verified by colony PCR and sequencing.

The recombinant plasmids pRSF-*cfa*1 and pRSF-*cfa*2 were transformed into *E. coli* BL21 (DE3) respectively to obtain strains *E. coli* BL101 (pRSF-*cfa*1) and *E. coli* BL102 (pRSF-*cfa*2). Similarly, the empty vector pRSFDuet-1 was also transformed into *E. coli* BL21 (DE3) to obtain a control strain *E. coli* BL100 (pRSFDuet-1). All transformants were screened on appropriate antibiotic resistance plates.

### CRISPR/Cas9-mediated genome editing and plasmid curing

Following the method of Zaigao Tan et al. (Tan et al., 2016), the *Accfa*2 gene was integrated into the genome of *E. coli* MG1655 using the CRISPR-Cas9 dual-plasmid system. The specific steps are as follows: Firstly, primer pairs Up_500_-F and Up_500_-R, Down_500_-F and Down_500_-R were used to amplify the upstream and downstream homologous arms at the *ldhA* target on the *E. coli* MG1655 genome by PCR, and the amplified products were named Up_500_ and Down_500_, respectively (It is worth noting that, a BT5 terminator sequence was introduced in Up_500_ to eliminate the influence of endogenous promoter of *ldhA* gene (Hao et al., 2020)). Meanwhile, using the genomic DNA of *A. caldus* CCTCC AB 2019256 as a template, primer pairs M1-93-F and M1-*cfa*2-R, M1-37-F and M1-*cfa*2-R, M1-12-F and M1-*cfa*2-R were used to amplify the *Accfa*2 gene, and the amplified products were named M1-93-*cfa*2, M1-37-*cfa*2 and M1-12-*cfa*2, respectively. The above PCR products were purified, and the *Accfa*2 gene, regulated by three different promoters with varying strengths, was subsequently assembled individually with Up_500_ and Down_500_ by overlapping extension PCR, and the assembled DNA products were inserted into pRSFDuet-1 for storage. Three linear template fragments were amplified from the recombinant plasmids using primer pairs Linear-*cfa*2-HF-F and Linear-*cfa*2-HF-R, Then, each purified PCR product (400 ng) was co-transformed by electroporation together with 100 ng pTargetF-Δ*ldhA* into *E. coli* MG1655 competent cells harboring pCas plasmid. The cells were spread on LB plates containing 50 μg/ml spectinomycin and 50 μg/ml kanamycin, incubated overnight at 30 °C, and three *Accfa*2 gene knock-in mutants (M1-93-*Accfa*2, M1-37-*Accfa*2, and M1-12-*Accfa*2) were obtained after sequencing verification.

Plasmid curing was carried out as reported by Yu Jiang et al. (Jiang et al., 2015). Briefly, the first was the cure of the pTarget series plasmids. The bacteria were inoculated in LB medium supplemented with kanamycin (50 μg/ml) and 0.4 mM isopropyl-*β*-d-thiogalactopyranoside (IPTG) and cultivated overnight at 30 °C, the sensitivity of cells to spectinomycin was used to determine whether the pTargetF-Δ*ldhA* in the colonies was successfully cured. Then came the cure of the temperature-sensitive pCas plasmid. The pTarget-cured bacteria were cultivated overnight at 37 °C, and the sensitivity of cells to kanamycin was used to determine whether the pCas plasmid in the colonies was successfully cured.

### Determination of growth performance and kinetics

For the cell growth assay, the overnight cell cultures were diluted to an initial OD_600_ of 0.05 in fresh E minimal medium and incubated in an incubator shaker at 37 °C and 200 rpm until stationary phase. For strains with plasmid, IPTG with a final concentration of 0.4 mM was added when their OD_600_ reached 0.4, while the temperature was adjusted to 28 °C and the incubation was continued until stationary phase. The OD_600_ of all samples were measured using a uv/vis spectrophotometer (UV-5200, METASH). The specific growth rate of bacteria was calculated as follows. Where $\mu_{x}$ is the specific growth rate, $X_{0}$ and $X_{t}$ represent the initial biomass and the biomass at the time of detection, respectively.

$$\mu_{x}=\ln\left(X_{t}/X_{0}\right)/t$$

### Analysis of cell membrane fatty acid composition and membrane properties

Phospholipids were extracted as described by Feng et al. (Feng et al., 2015). Briefly, *E. coli* cells with a wet weight of 0.2 g were harvested, 2.5 ml 1 M NaOH-methanol solution was added, and the cells were shaken and resuspended. The suspension was transferred to a 10 ml colorimetric tube, and the reaction was heated in a water bath at 70 °C for 1 h. After cooling at room temperature for 10 min, 2.5 ml of 25% boron trifluoride diethyl ether-methanol solution was added, and esterifying for 20 min in a 65 °C water bath. After cooling at room temperature for 10 min, added 2 ml of n-hexane, vortex for 1 min, then added 2 ml of saturated NaCl solution, vortex for 1 min, and then centrifuged at 3000 rpm for 1 min drew the upper phase, treated with a small amount of anhydrous Na_2_SO_4_ to remove water (about 1–2 h), after standing, the upper phase was drawn and passed through a 0.22 μm organic filter membrane, and the solution was collected into a 2 ml gas chromatography injection vial for testing. The fatty acid composition of the samples was analyzed by a trace GC/MS instrument (Varian, California, USA), and the relative percentage of each fatty acid was determined based on the peak area.

The cell membrane fluidity was assessed by measuring the fluorescence polarization value (negatively correlated with fluidity) of the 1, 6-Diphenyl-1,3, 5-Hexatriene (DPH) probe embedded in the lipid bilayer of the membranes (Wu et al., 2012). Briefly, cells before and after acid-treated were harvested, washed twice with phosphate-buffered saline (PBS, pH 7.0), resuspended (1×10^8^ cells/ml) and incubated in the dark at 37 °C for 30 min with DPH at a final concentration of 0.2 μM. Fluorescence polarization values were determined by using a spectrofluorometer (model 650–60, Hitachi) with excitation and emission wavelengths of 360 nm and 432 nm, respectively, the slit width of both excitation and emission light was 5 nm. The polarization ( p ) value is calculated according to the following formula:

$$p=\frac{I_{VV}-I_{VH}\left(I_{HV}/I_{HH}\right)}{I_{VV}+I_{VH}\left(I_{HV}/I_{HH}\right)}$$

In the formula, the subscripts V and H represent the vertical and horizontal orientations, respectively. For example, $I_{VH}$ represents the fluorescence intensity measured when the optical gratings of the excitation and analyzer polarizer are in the vertical and horizontal orientations, respectively, while $I_{HV}$ represents the fluorescence intensity measured when the optical gratings of the excitation and analyzer polarizer are in the horizontal and vertical orientations, respectively.

The cell membrane permeability was analyzed by measuring the fluorescence intensity of cells labeled with fluorescein diacetate (FDA) using the fluorescence spectrofluorometer (F-7000, Hitachi, Japan) based on the reference methods (Shen et al., 2011). The specific procedure was as follows: mixed 5 ml cell suspension (cell density reached 10^6^ cells/mL) with 625 μL FDA acetone solution (2 mg/ml), and the cell fluorescent intensity was examined after shaking in the dark at 32 °C for 5 min. The maximal excitation wavelength for FDA was 492 nm and the emission wavelength was 513 nm. The slit width of excitation wavelength and emission wavelength was 2.5 and 5 nm, respectively. The relative cell surface hydrophobicity measurements of the organisms were performed using the Microbial adhesion to hydrocarbon (MATH) method (Aono and Kobayashi, 1997).

### Examination of cell morphology

The *E. coli* cells cultured to the mid-exponential phase were collected by centrifugation, and after being subjected to acid stress (the cells were resuspended in E minimal medium with pH 4.2 for 2 h, followed by centrifugation at 12,000 *g* for 10 min), they were fixed with 2.5% (v/v) glutaraldehyde for 0.5 h, and then the samples were processed according to the procedure used in our previous study (Feng et al., 2021). Finally, the treated samples were observed using a transmission electron microscopy (Hitachi-H 7000, Hitachi, Japan).

### Analysis of intracellular free amino acids

For the analysis of intracellular free amino acids, *E. coli* cells cultured to the mid-exponential phase with a wet weight of approximately 0.2 g were collected by centrifugation (When needed, the treatment method of acid stress was the same as above), the bacterial pellet was washed twice with PBS (pH 7.0), resuspended in 1 ml of 5% (w/v) trichloroacetic acid solution, then sonicated at room temperature for 20 min and allowed to stand for 2 h. After centrifugation at 12,000 *g* for 10 min to remove cell debris, the supernatant was filtered through a 0.22 μm filter membrane and then the free amino acids in the samples were determined by HPLC (Agilent 1200, Agilent, USA) according to the method of Fountoulakis et al. (Fountoulakis and Lahm, 1998).

### Determination of intracellular ATP content and intracellular pH

The *E. coli* cells cultured to the mid-exponential phase were collected by centrifugation. The bacteria particles were resuspended in the E minimal medium with pH 4.2 and incubated for 0.5 h, 1 h and 2 h, respectively. Subsequently, the cells were washed with prechilled PBS (pH 7.0) and lysis buffer associated with the Enhanced ATP Assay Kit, S0027 (Beyotime Biotechnology, Shanghai, China) was used for cell lysis. The same procedure was performed on cells that had not been treated with acid stress. The content of intracellular ATP (nmol/mg protein) was determined by using the above-mentioned kit according to the manufacturer’s instructions, and the associated protein concentrations were determined by Coomassie Blue staining.

The pH sensitive dye 2′, 7′-bis-(2-carboxyethyl)-5-(and-6) carboxyfluorescein, acetoxymethyl ester (BCECF AM; Beyotime, China) was used for intracellular pH (pH_in_) detection, and the treatment method of acid stress was the same as above. The specific steps were as follows: First, the harvested *E. coli* cells cultured to the mid-exponential phase were washed and resuspended with 50 mM HEPES-K buffer (pH 8.0). Next, BCECF AM with a final concentration of 1 μM was added and mixed, and incubated at 30 °C in the dark for 20 min. Subsequently, the cells were washed thrice with 50 mM potassium phosphate buffer (pH 7.0) and resuspended, and the fluorescence intensity of the bacterial suspension $\left(I_{total}\right)$ and the supernatant $\left(I_{filtrate}\right)$ were measured using a fluorescence spectrophotometer (F-7000). The excitation wavelength was set at 488 nm and 440 nm, while the emission wavelength was 525 nm and the slit width was 5 nm. The formula for calculating fluorescence intensity was as follows:

$$I=\frac{{\left(I_{490}\right)}_{total}-{\left(I_{490}\right)}_{filtrate}}{{\left(I_{440}\right)}_{total}-{\left(I_{440}\right)}_{filtrate}}$$

### Characterization of strain tolerance

All tolerance experiments were performed in 50 ml E minimal medium in 250 ml baffled flasks at 200 rpm and initial pH of 7.0. Except for the high temperature tolerance and octanoic acid tolerance experiments, which were evaluated at 42 °C and 30 °C respectively, the rest of the tolerance experiments were conducted at 37 °C.

### Statistical analysis

All experiments were carried out in triplicate. One-way ANOVA and student’s t-test were used to evaluate statistical differences between the groups of experimental data, and *p* < 0.05 was considered statistically significant.

## Results

### Effect of *Accfa* overexpression in *E. coli* on its acid resistance

With reference to the whole genome sequence of a model strain *A. caldus* ATCC 51756 submitted to NCBI, we cloned two CfaS coding genes (*cfa*1 and *cfa*2) from the genomic DNA of strain *A. caldus* CCTCC AB 2019256 preserved in our laboratory. After sequencing verification, it was found to have 100% homology with the two *cfa* gene sequences of the published model strain *A. caldus* ATCC 51756. Subsequently, we transformed the constructed recombinant plasmids (pRSF-*cfa*1 and pRSF-*cfa*2) into *E. coli* BL21 (DE3) and tested the growth performance of the recombinant strains using E minimal medium.

**FIGURE 1:**
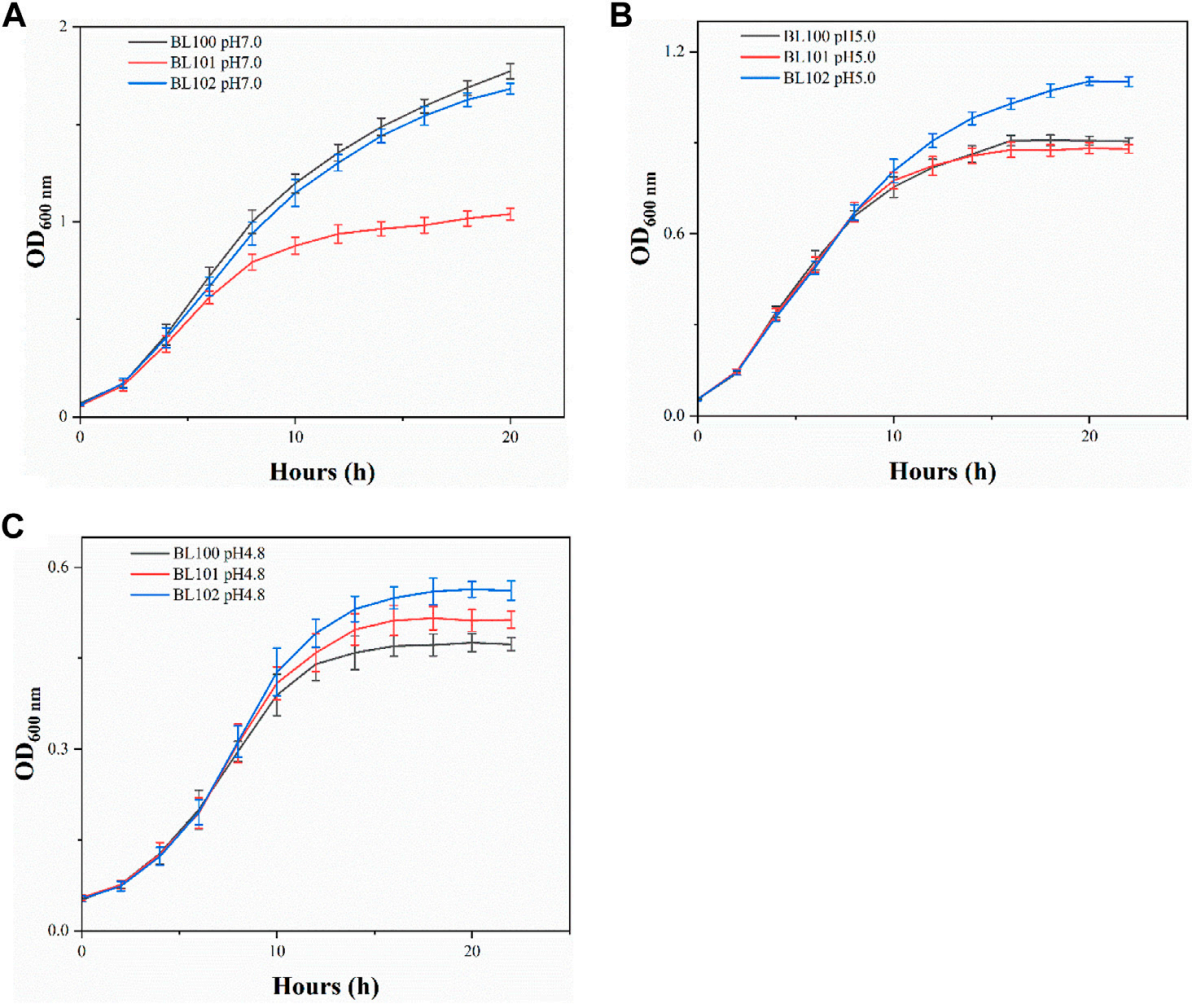
The overexpression of *cfa*2 from *A. caldus* improved the acid resistance of *E. coli*. **(A**–**C)** The growth curves of strains BL100-102 cultivated in E minimum medium with pH 7.0, pH 5.0 and pH 4.8, respectively. The error bars represented means ± standard deviations (SD).

When incubated at pH 7.0, each overexpression strain had lower cell concentrations than the control strain BL100 throughout the fermentation (Figure 1A). Strains BL100 and BL101 had entered the stationary phase after 16 h incubation at pH 5.0. In contrast, the biomass of strain BL102 continued to increase, indicating that the overexpression of *cfa*2 had superior resistance to acid stress. After 20 h, the OD_600_ of the strain BL102 increased by 21.6% compared to the control (Figure 1B). As for pH 4.8, the biomass of strains BL101 and BL102 increased to varying degrees, and after incubation for 18 h, their OD_600_ increased by 9.3% and 18.7%, respectively, compared with the control (Figure 1C).

### Integration of *Accfa*2 into the genome via CRISPR-Cas9 improved the acid resistance of *E. coli*

**FIGURE 2:**
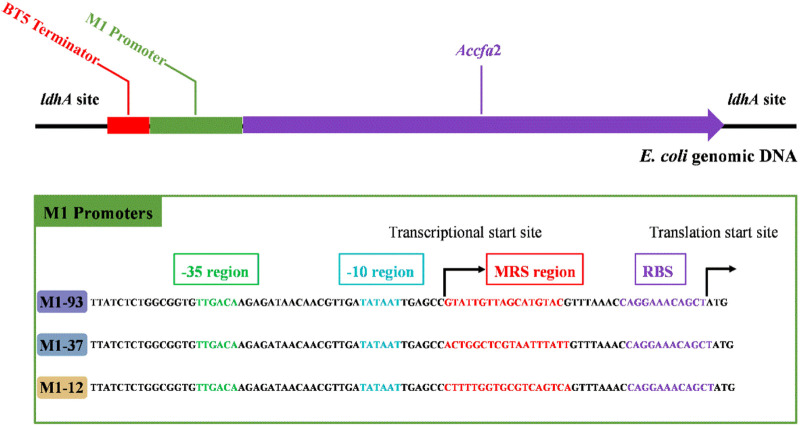
Integration of *cfa*2 from *A. caldus* CCTCC AB 2019256 into genomic DNA of *E. coli* MG1655 at the *ldhA* (lactate dehydrogenase) site using the CRISPR-Cas9 dual-plasmid system. A BT5 terminator was introduced to eliminate the influence of endogenous promoter of the *ldhA* gene. The three different promoters employed to regulate *cfa*2 expression have the same -35 region, -10 region and RBS region, while the mRS (mRNA stabling) region is distinct. See reference 4 for details on promoters.

Given the excellent acid resistance of *E. coli* BL21 brought by the overexpression of the *cfa*2 gene, simultaneously, for the purpose of eliminating the disadvantages (such as the use of expensive antibiotics, the instability of gene expression, and the difficulty in keeping synchronization between plasmid replication and cell division, *etc.*) caused by plasmid overexpression, we then used the CRISPR-Cas9 dual-plasmid system to construct a series of knock-in mutants on the genome of *E. coli* MG1655 (Figure 2), in which *cfa*2 was regulated by three different promoters with varying strengths (M1-93, M1-37, M1-12), and the growth performance and kinetics of the mutants were tested.

**FIGURE 3:**
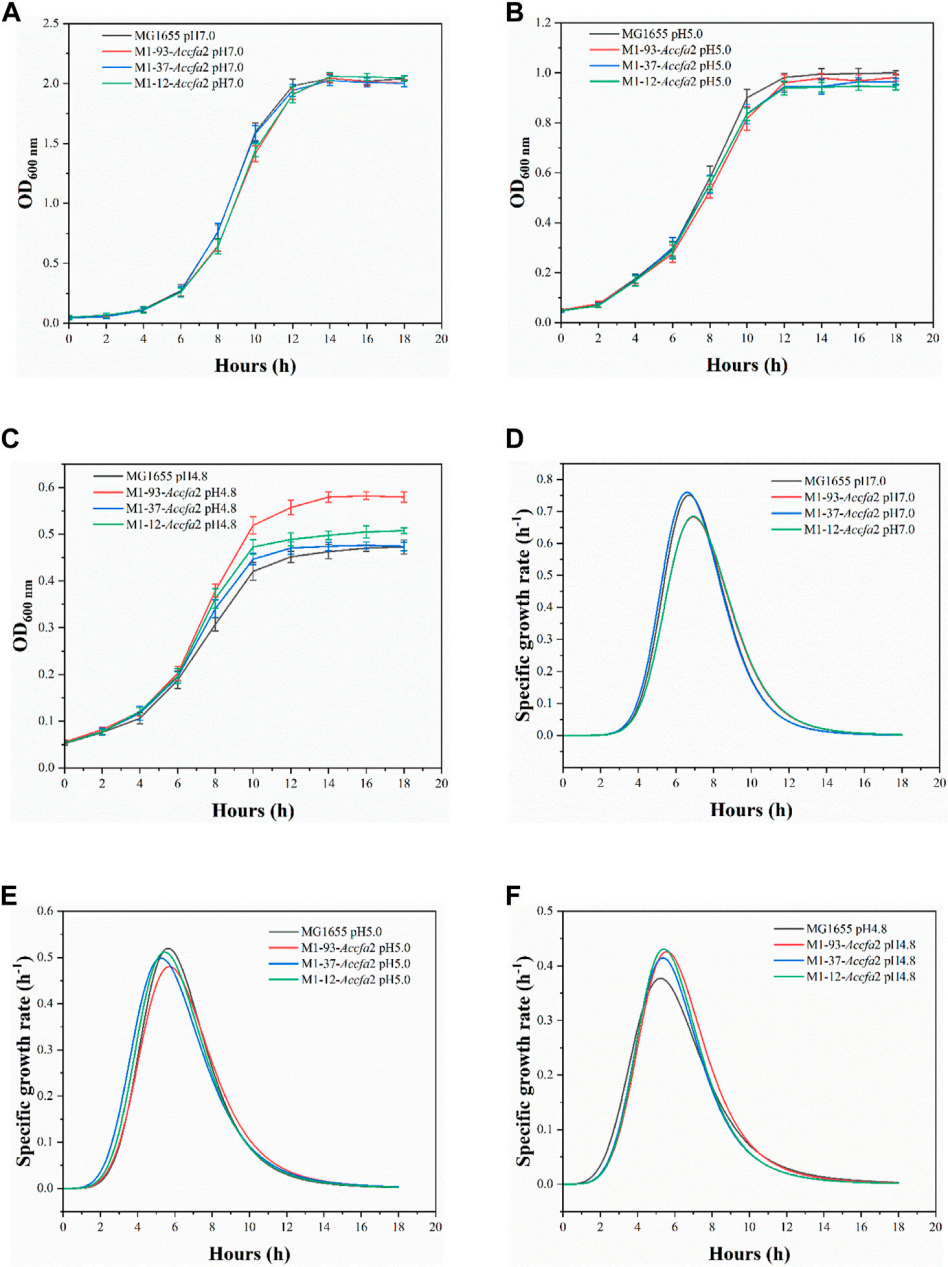
The integration of *Accfa*2 into genomic DNA of *E. coli* increased its resistance to severe acid stress (pH 4.8). **(A**–**C)** The growth curves of *E. coli* under neutral (pH 7.0), moderate (pH 5.0) and severe acid stress (pH 4.8) conditions, respectively, and E minimal medium was used for incubation. **(D**–**F)** The specific growth rate curves corresponding to the above three conditions, respectively. The error bars represented means ± standard deviations (SD).

Compared with MG1655, the mutants showed no growth advantage under neutral (pH 7.0) and moderate acid stress (pH 5.0) conditions (Figures 3A, B). However, when incubated under severe acid stress (pH 4.8), the mutants exhibited a clear growth advantage. Especially for M1-93-*Accfa*2, after incubation for 10 h and 14 h, its OD_600_ increased by 23.4% and 25.3% compared with the control strain, respectively (Figure 3C). Consistently, under severe acid stress condition, the maximum specific growth rate of the mutants maintained in the range of 0.415–0.431 h^−1^, while this value of the control strain decreased to 0.377 h^−1^ (Figure 3F). Nevertheless, the maximum specific growth rate of the mutants was not superior to that of the control strain under the other two conditions (Figures 3D, E).

### Changes in membrane related properties caused by the heterologous expression of *Accfa*2

**FIGURE 4:**
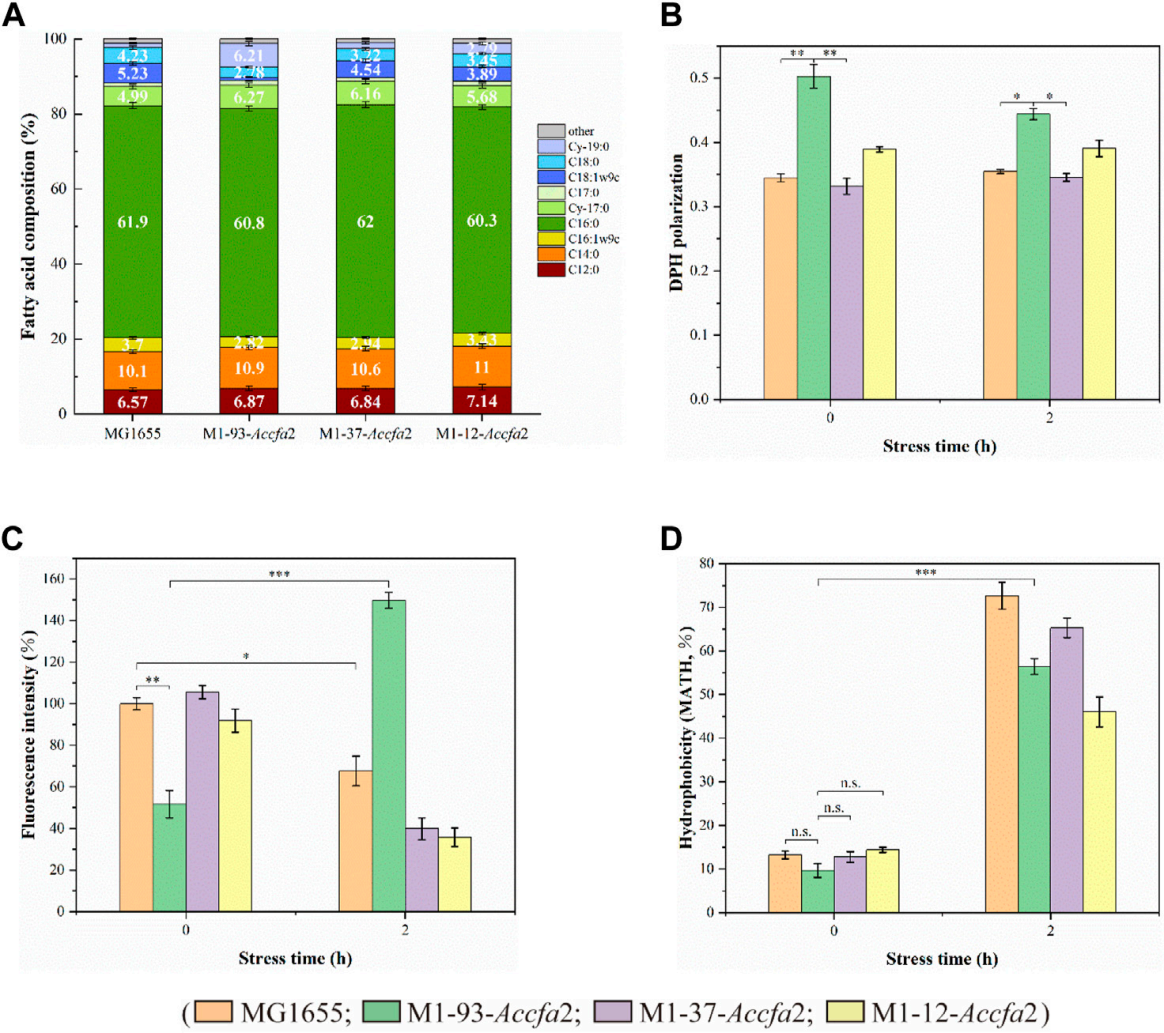
The heterologous expression of *Accfa*2 altered the membrane properties of *E. coli*. **(A)** The fatty acid profiles of strains MG1655, M1-93-*Accfa*2, M1-37-*Accfa*2 and M1-12-*Accfa*2 cultivated to the exponential phase in E minimal medium. **(B**–**D)** The alterations in cell membrane fluidity, permeability and relative hydrophobicity of cell surface of the above four strains before and after exposure to acid stress, respectively. The error bars represented means ± standard deviations (SD).

We expected that heterologous expression of *Accfa*2 would affect membrane properties by altering the levels of CFAs in the cell membrane, which could provide a basis for the improved acid resistance. Indeed, compared with the control strain, the proportions of two CFAs (Cy-17:0 and Cy-19:0) in the cell membrane of the mutants were increased to varying degrees. In particular, for M1-93-*Accfa*2, Cy-17:0 and Cy-19:0 accounted for 6.27% and 6.21% of the cell membrane fatty acid profile, respectively, which were 1.26 and 5.26 times higher than that of the control strain (4.99% and 1.18%). Correspondingly, as a precursor for the synthesis of CFAs, the proportion of UFAs in the cell membrane of the mutants decreased to varying degrees, among which the two UFAs, C16:1w9c and C18:1w9c, in the cell membrane of M1-93-*Accfa*2 accounted for 2.82% and 0.90%, respectively, which decreased by 1.31 and 5.81 times compared with 3.70% and 5.23% of the control strain (Figure 4A).

**FIGURE 5:**
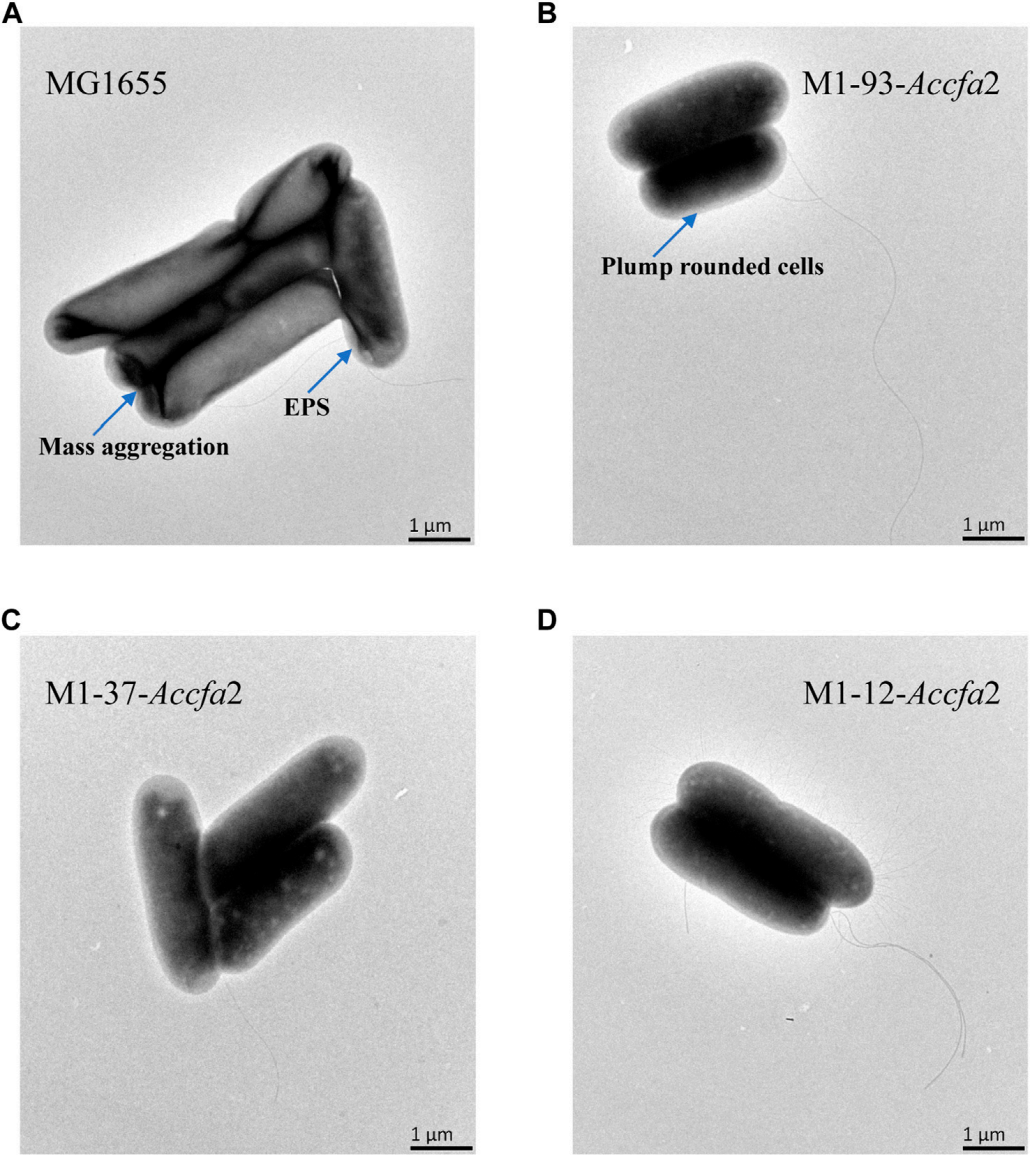
The morphological characteristics of strains MG1655 **(A)**, M1-93-*Accfa*2 **(B)**, M1-37-*Accfa*2 **(C)** and M1-12-*Accfa*2 **(D)** treated with acid stress.

We further investigated the effects of increased proportions of CFAs on the cell membrane fluidity, permeability, and the cell surface hydrophobicity. Membrane fluidity was determined by analyzing the fluorescence anisotropy of 1, 6-diphenyl-1,3,5-hexatriene (DPH), which is negatively correlated with fluidity. The cell membrane fluidity of the mutants M1-93-*Accfa*2 and M1-12-*Accfa*2 was significantly lower than that of the control strain, especially M1-93-*Accfa*2, but the differences among the strains tended to decrease after environmental acidification (Figure 4B). The permeability of the cell membrane is quantified by measuring the fluorescence intensity generated by FDA cleavage that has penetrated into the cell. When no acid stress was imposed, the mutant M1-93-*Accfa*2 had the lowest cell membrane permeability (roughly half that of the control strain). After 2 h of acid stress treatment, the permeability of M1-93-*Accfa*2 increased approximately 2-fold, whereas other strains exhibited varying degrees of decrease (Figure 4C). The results of the relative hydrophobicity test on the cell surface indicated that, although the differences were not significant, the hydrophobicity of the mutant M1-93-*Accfa*2 was still the lowest when no acid stress was imposed, and the hydrophobicity of each strain increased remarkably after environmental acidification (Figure 4D). The morphological observation of cells treated with acid stress by transmission electron microscopy (TEM) demonstrated that the cells of the mutants, especially M1-93-*Accfa*2, were able to maintain a plump external morphology, while the aggregation of the control strain was intensified and the cells were more elongated (Figures 5A–D).

### Changes in intracellular amino acid metabolism and ATP levels caused by the heterologous expression of *Accfa*2

**FIGURE 6:**
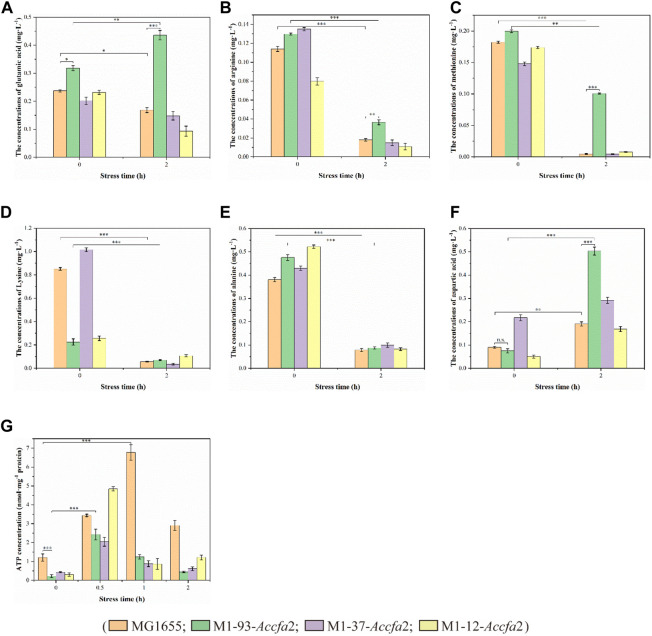
The physiological effects caused by the heterologous expression of *Accfa*2 on the acid resistance mechanism of *E. coli*. **(A**–**F)** The alterations in the concentrations of intracellular glutamic acid, arginine, methionine, lysine, alanine and aspartic acid in the strains MG1655, M1-93-*Accfa*2, M1-37-*Accfa*2 and M1-12-*Accfa*2 before and after exposure to acid stress, respectively. **(G)** The alteration in the concentrations of intracellular ATP in the strains mentioned above.

Previous studies have shown that amino acid metabolism can assist microorganisms resist acidic environments, and its role in microbial cell acid resistance has been repeatedly confirmed by microenvironment level and proteomic analysis (Len et al., 2004; Lu et al., 2013). Therefore, we subsequently investigated the changes in intracellular free amino acids content of the above four strains before and after exposure to acid stress. After 2 h of acid stress treatment, the intracellular contents of several amino acids (such as glutamic acid, arginine, methionine, lysine, and alanine etc.) closely related to acid resistance of *E. coli* presented a clear downward trend (Figures 6A–E), on the contrary, the intracellular content of aspartic acid presented an upward trend (Figure 6F). It should be noted that after exposure to acid stress for 2 h, the content of glutamic acid in mutant M1-93-*Accfa*2 cells increased approximately 1.37-fold compared with that before stress treatment (Figure 6A). Meanwhile, in contrast, the intracellular contents of glutamic acid, arginine, methionine and aspartic acid of M1-93-*Accfa*2 were the highest, which were 2.59, 2.04, 22.07 and 2.65 times of the control strain, respectively (Figures 6A–C, F). It has been reported that cyclopropanation requires the consumption of a metabolically expensive intermediate named AdoMet (three ATPs are expended in the synthesis of each AdoMet molecule) (Chang and Cronan, 1999; Chang et al., 2000), in line with this, our subsequent tests showed that the intracellular ATP content of the three mutants was much lower than that of the control strain. After 0.5 h of acid stress treatment, the intracellular ATP content of each strain increased remarkably, however, with the extension of stress time, the intracellular ATP content of each strain demonstrated an evident downward trend (Figure 6G). Subsequently, the intracellular pH was monitored using the fluorescent probe BCECF AM, and the results demonstrated that there was no significant difference observed among the strains before and after exposure to acid stress despite the varied CFA contents in membrane.

### Heterologous expression of *Accfa*2 enhanced stress resistance of *E. coli* to succinic acid

**FIGURE 7:**
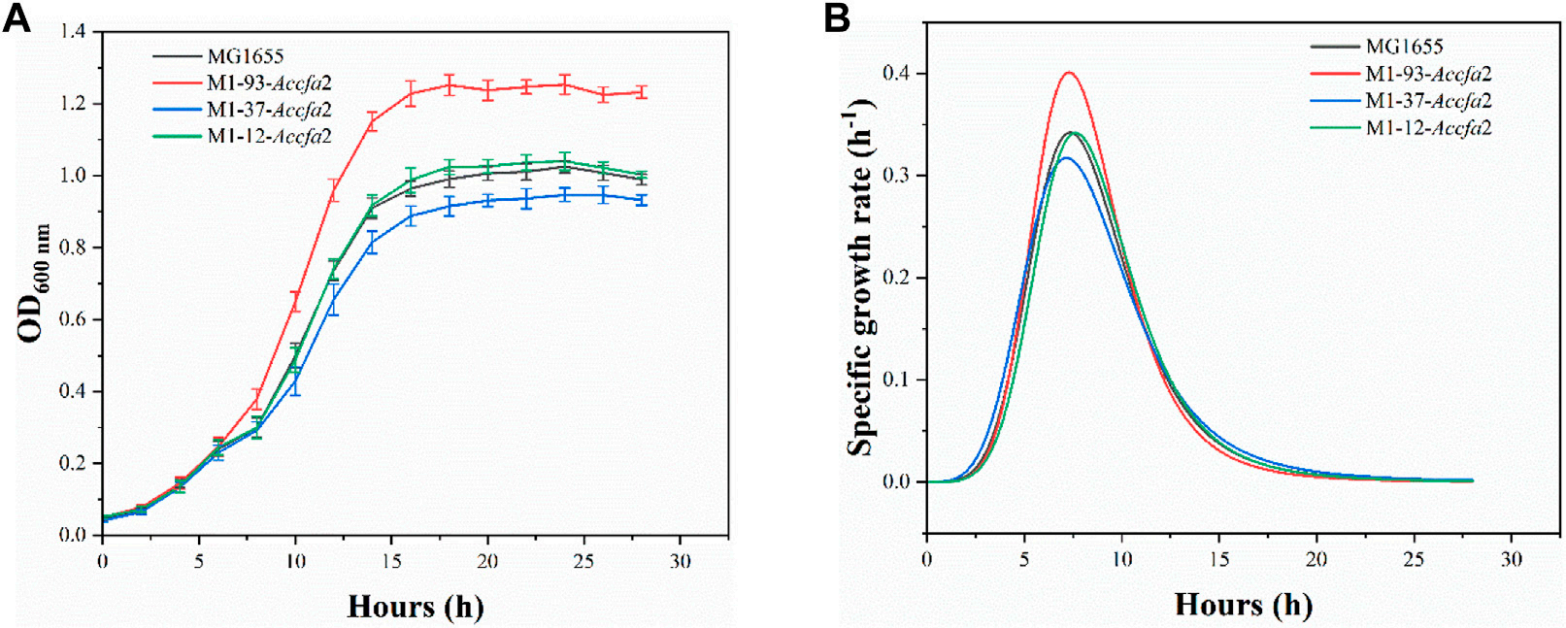
The heterologous expression of *Accfa*2 enhanced the resistance of *E. coli* to succinic acid. **(A)** The growth curves of the strains MG1655, M1-93-*Accfa*2, M1-37-*Accfa*2 and M1-12-*Accfa*2 in the presence of succinic acid at a final concentration of 40 mM. **(B)** The specific growth rate curves fitted according to the growth curves. The error bars represented means ± standard deviations (SD).

Given that heterologous expression of *Accfa*2 altered the membrane properties of engineered bacteria, we subsequently investigated whether this CfaS-based membrane engineering strategy has a general effect of improving microbial robustness in the context of other inhibitory compounds and/or harsh processing conditions. Four general classes of compounds were considered, organic acids, short-chain fatty acids, alcohols, and aromatics, as well as two classes of unfavorable processing conditions, high temperature and high osmotic pressure. The concentrations of the inhibitors were selected with reference to previous reports (Tan et al., 2016). It was found that the engineered strain M1-93-*Accfa*2, which performed well under severe acid stress, also exhibited apparent growth advantages against organic acids, especially succinic acid (data for other chemical and environmental challenges were shown in Supplementary Figure S2). Specifically, after incubation for 16 h in E minimal medium with a final concentration of 40 mM succinic acid, its OD_600_ increased by 27.4% compared with the control strain, and the maximum specific growth rate was also increased from 0.342 h^−1^ of the control strain to 0.401 h^−1^ (Figures 7A, B).

## Discussion

Two CfaS coding gene sequences (*cfa*1 and *cfa*2) were easily cloned from the genome of *A. caldus* CCTCC AB 2019256 with designed primers due to the high homology with the sequences of the model strain *A. caldus* ATCC 51756. Furthermore, since the overexpression of *cfa*2 gene endowed *E. coli* with high-performance on resistance to the acid stress, we used *cfa*2 to construct subsequent knock-in mutants. In order to minimize the interference to adjacent genes, we did not delete the complete *ldhA* gene when integrating *cfa*2 into the *ldhA* site on the *E. coli* genome (because the N-terminal sequence of the *ldhA* gene overlaps slightly with the promoter sequence of the neighboring *ydbH* gene). In addition, it was found through pre-experiment that *E. coli* could not grow in E minimal medium with pH 4.2, even if it was pre-adapted at pH 5.0. Therefore, we chose E minimal medium with pH 4.2 as the acid stress condition for the subsequent studies. Meanwhile, although pH 5.0 and pH 4.8 were relatively close in value, the final biomass of *E. coli* cultured at pH 4.8 was almost 50% lower than that at pH 5.0 (Figures 1B, C; Figures 3B, C). Therefore, compared with moderate acid stress (pH 5.0), we defined pH 4.8 as severe acid stress in this paper (Xu Y. et al., 2020).

**FIGURE 8:**
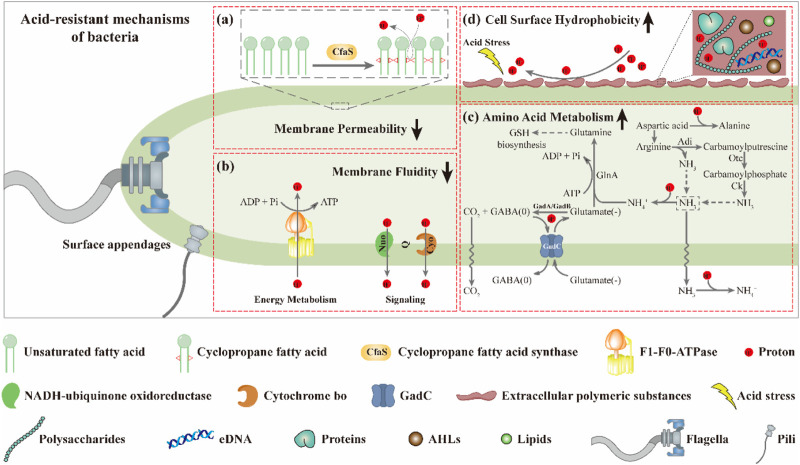
The CfaS-mediated cell membrane remodeling favors bacterial resistance to environmental acid stress. The heterologous expression of *Accfa*2 led to a significant decrease in the membrane permeability **(A)** and fluidity **(B)** of bacteria. Under acid stress, the intracellular amino acid metabolism of bacteria was enhanced **(C)**, and the relative hydrophobicity of the cell surface was increased **(D)**.

Several studies have shown that the fluidity of the cell membrane is positively correlated with the proportion of UFAs in the membrane (Fang et al., 2017; Fernández et al., 2019; Breton et al., 2020; Resemann et al., 2021). Based on this, it is not surprising that the membrane fluidity of the mutants M1-93-*Accfa*2 and M1-12-*Accfa*2 observed here was lower than that of the control strain (Figure 4B). That is, in the cells of M1-93-*Accfa*2 and M1-12-*Accfa*2, a considerable proportion of UFAs were consumed due to the synthesis of CFAs (Figure 4A), which led to a decrease in membrane fluidity. Not only membrane fluidity, but also membrane permeability measurements of each strain could be interpreted according to the fatty acid profiles. That is, when no acid stress was imposed, the mutant M1-93-*Accfa*2 had the lowest cell membrane permeability (Figure 4C), which was closely associated with the highest expression intensity of *Accfa*2 and the highest proportion of CFAs synthesis. It has been reported that CpxRA, a two-component system of *E. coli*, directly senses acidification through protonation of CpxA periplasmic histidine residues, and upregulates two desaturase-encoding genes, *fabA* and *fabB*, resulting in increased production of UFAs (Xu Y. et al., 2020). Therefore, after 2 h of acid stress treatment, the abundance of UFAs in the cell membrane might be up-regulated, leading to a significant increase in membrane permeability (Figure 4C). Fluorescein diacetate (FDA) used to detect the membrane permeability here needs to be catalyzed by intracellular esterase to produce green fluorescence, and the fluorescence could only be maintained within the intact membrane (Aeschbacher et al., 1986; Dive et al., 1988). Therefore, the reason for the decreased membrane permeability of the other strains might be that a considerable proportion of the bacteria had died due to the damaged of the cell membrane under the effect of acid stress (Figure 4C). As for the reason why the relative hydrophobicity on the cell surface of each strain increased dramatically after being exposed to acid stress (Figure 4D), it was speculated that substantial extracellular polymeric substances (EPS) were secreted from the bacteria cells (Flemming et al., 2016). The results observed by TEM are consistent with this, i.e., the generation of EPS promoted the aggregation of cells (Figure 5A). In summary, in the cells of the engineered strain, especially M1-93-*Accfa*2, the heterologous expression of *Accfa*2 led to a significant decrease in its membrane permeability and fluidity. Meanwhile, the relative hydrophobicity of the cell surface increased under the stimulation of environmental acid stress (Figures 8A, B, D).

The process of microbial resistance to environmental stress typically consumes intracellular energy (Krulwich et al., 2011). We observed that the intracellular ATP content of each strain increased significantly at the initial stage of acid stress (Figure 6G), which might be because the bacteria strengthened ATP synthesis in various metabolic pathways to resist the acid stress suddenly imposed in the external environment. With the extension of stress process, the intracellular ATP content of each strain decreased to varying degrees, which may be that the bacterial cells initiated other mechanisms to resist acid stress, resulting in a considerable proportion of ATP being consumed (Figure 6G). Similarly, after exposure to acid stress, the reduction in the content of intracellular free amino acids such as glutamate and arginine could be attributed to the substantial consumption in the acid-resistant reactions (such as decarboxylation) (Liu et al., 2015). Among them, the glutamate-dependent acid resistance (AR) system has been shown to be the most effective system for alleviating acid stress in *E. coli* (Seo et al., 2015). The specific mechanism can be summarized as follows. Glutamate (-) was converted to GABA (0) with decarboxylases (GadA and GadB), and one proton was consumed at the same time. Glutamate (-) was subsequently imported from the extracellular *via* a transmembrane antiporter (GadC) in exchange for the GABA (0) so that the reaction continued (Hu et al., 2020) (Figure 8C). It has been reported that aspartic acid could be converted into alanine to consume intracellular protons. Meanwhile, aspartic acid could also be converted into arginine to enter the ADI pathway to generate NH_3_ that could neutralize protons (Krulwich et al., 2011) (Figure 8C). Therefore, the intracellular reserve of aspartic acid was conducive to the response to acid stress. As the engineered strain M1-93-*Accfa*2 had the highest intracellular content of several amino acids associated with acid resistance compared with other strains, it was not surprising that it exhibited the highest-performance on resistance to the acid stress.

Microbial acid resistance is a complex polygenic trait involving multiple regulatory systems (Lin et al., 2021). Due to the possible compensatory effect among different systems (Pando et al., 2017), there was no significant difference in intracellular pH among the strains detected in this study. Cyclopropanation consumes a metabolically expensive intermediate, S-adenosine methionine (AdoMet), which is required for the methylation of nucleic acids and the synthesis of other substances such as spermidine (Chang et al., 2000). Furthermore, since the M1 promoter used in this study was a constitutive type, the *Accfa*2 gene would be continuously expressed in the cell. When using a plasmid to overexpress the *Accfa*2 gene, a similar effect will be produced after adding IPTG, as it is not metabolized by the cells. In this way, AdoMet, which is already in short supply in the cell, is continuously consumed, which might be the reason why the heterologous expression of *Accfa* gene in *E. coli* did not show a growth advantage under neutral or moderate acid stress condition. Conversely, when the environmental pH drops to a life-and-death level for the bacteria, the consumption of intracellular AdoMet is worthwhile relative to the survival of the bacteria from the perspective of cell metabolism, which might be the reason why the heterologous expression of *Accfa* in *E. coli* showed a growth advantage under severe acid stress condition.

When engineered bacteria are applied to industrial production as cell factories, they often face problems such as plasmid compatibility and metabolic burden (Zhang et al., 2019). So here we first evaluated the effects of *cfa*1 and *cfa*2 genes on improving the acid resistance of *E. coli* through plasmid overexpression, and on this basis, constructed a series of knock-in mutants of *Accfa*2 gene. In this way, while retaining the endogenous *cfa* gene, an additional copy of the *cfa* derived from extreme acidophile was added to the genome of *E. coli*. Since CfaS exerts different effects on different molecules (Choi T. R. et al., 2020), the role of the cell membrane as a defensive barrier might be strengthened. Although the engineered strains constructed here did not exhibit growth advantage (no significant difference) under neutral and moderate acid stress conditions, it is conceivable that M1-93-*Accfa*2 will exhibit obvious growth advantage when the pH of fermentation broth continues to decline. Obviously, a certain scale of viable bacteria count is the premise of maintaining a stable production capacity. Therefore, this study demonstrated the potential of improving the acid resistance of engineered strains for industrial production.

## Data Availability

The original contributions presented in the study are included in the article/Supplementary Material, further inquiries can be directed to the corresponding authors
