# Supplementary material for: Improving acid resistance of Escherichia coli base on the CfaS-mediated membrane engineering strategy derived from extreme acidophile
Source: Front Bioeng Biotechnol. 2023 Mar 21;11:1158931. doi: 10.3389/fbioe.2023.1158931 (PMC10070827; doi:10.3389/fbioe.2023.1158931)
Supplement: Supplementary file 1 [file DataSheet1.docx]

**Supplementary Material**

**Journal: Frontiers in Bioengineering and Biotechnology**

**Title:** **Improving Acid Resistance of *Escherichia coli* Base on the CfaS-mediated Membrane Engineering Strategy Derived from Extreme Acidophile**

Wenbo Hu^1^, Yanjun Tong^2^, Junjie Liu^1^, Panyan Chen^3^, Hailin Yang^1,3*^ and Shoushuai Feng^1,3*^

^1^ The Key Laboratory of Industrial Biotechnology, Ministry of Education; School of Biotechnology, Jiangnan University, Wuxi, 1800 Lihu Road, People’s Republic of China;

^2^ State Key Laboratory of Food Science and Technology, Jiangnan University, Wuxi, People’s Republic of China;

^3^ Key Laboratory of Carbohydrate Chemistry and Biotechnology (Jiangnan University) Ministry of Education, Jiangnan University, Wuxi, 1800 Lihu Road, People’s Republic of China.

🖂 Shoushuai Feng

E-mail: [fengss@jiangnan.edu.cn](mailto:fengss@jiangnan.edu.cn)

Tel: +86 85913671.

Fax: +86 85918119.

**Supplementary Tables**

Supplementary Table S1. Strains and plasmids used in this study.

| Strains or plasmids | Genotype or description | Source |
| --- | --- | --- |
| Strains |  |  |
| *A. caldus* CCTCC AB 2019256 | Isolated from the AMD of Zijin Copper Mine, Fujian Province, China. | Lab stock |
| *E. coli* DH5α | F^–^ *deoR endA1 gyrA96 hsdR17*(rk^–^ mk^+^) *recA1 relA1 supE44 thi-1*  *Δ(lacZYA-argF)U169* Φ80d *lacZ*ΔM15 λ^–^ | Vazyme |
| *E. coli* BL21 (DE3) | F^–^ *ompT hsdS(rB^–^mB^–^) gal dcm* (DE3) | Vazyme |
| *E. coli* MG1655 | K-12 F^–^ λ^–^ *ilvG^–^ rfb-50 rph-1* | Lab stock |
| BL100 | *E. coli* BL21 (DE3) harboring pRSFDuet-1 | This study |
| BL101 | *E. coli* BL21 (DE3) harboring pRSF-*cfa*1 | This study |
| BL102 | *E. coli* BL21 (DE3) harboring pRSF-*cfa*2 | This study |
| M1-93-*Accfa*2 | The *cfa*2 gene from *A. caldus* which regulated by the M1-93 promoter was incorporated into the *ldhA* site on the genome of *E. coli* MG1655. | This study |
| M1-37-*Accfa*2 | The *cfa*2 gene from *A. caldus* which regulated by the M1-37 promoter was incorporated into the *ldhA* site on the genome of *E. coli* MG1655. | This study |
| M1-12-*Accfa*2 | The *cfa*2 gene from *A. caldus* which regulated by the M1-12 promoter was incorporated into the *ldhA* site on the genome of *E. coli* MG1655. | This study |
|  |  |  |
| Plasmids |  |  |
| pRSFDuet-1 | Double T7 promoters, RSF ori, Kan^r^ | Novagen |
| pRSF-*cfa*1 | pRSFDuet-1 carrying *cfa*1 | This study |
| pRSF-*cfa*2 | pRSFDuet-1 carrying *cfa*2 | This study |
| pTargetF-Δ*ldhA* | *pMB1* sgRNA-*ldhA* Spe^r^ | This study |
| pCas | *repA101*(Ts) Pcas-*cas9* ParaB-Red *lacI*^q^ Ptrc-sgRNA-*pMB1* Kan^r^ | Lab stock |

Supplementary Table S2. Primers used in this study.

| Primer name | Sequence (5' to 3')^a^ |
| --- | --- |
| pRSF-F1 | CTGCAGGTCGACAAGCTTGC |
| pRSF-R1 | GCTCGAATTCGGATCCTGGC |
| *cfa*1-F | CAGGATCCGAATTCGAGCATGAATACCGTTACCGCGACCC |
| *cfa*1-R | AAGCTTGTCGACCTGCAGTCACGCATGGCAGAGCGTCC |
| *cfa*2-F | CAGGATCCGAATTCGAGCATGAAAGACCTACGTTCTCATTTTCTCGAC |
| *cfa*2-R | AAGCTTGTCGACCTGCAGTCAAAGTTCGCCCCAATCGGG |
| M1-93-F | TTATCTCTGGCGGTGTTGACAAGAGATAACAACGTTGATATAATTGAGCCGTATTGTTAGCATGTACGTTTAAACCAGGAAACAGCTATGAAAGACCTACGTTCTCATTTTCTCGAC |
| M1-37-F | TTATCTCTGGCGGTGTTGACAAGAGATAACAACGTTGATATAATTGAGCCACTGGCTCGTAATTTATTGTTTAAACCAGGAAACAGCTATGAAAGACCTACGTTCTCATTTTCTCGAC |
| M1-12-F | TTATCTCTGGCGGTGTTGACAAGAGATAACAACGTTGATATAATTGAGCCCTTTTGGTGCGTCAGTCAGTTTAAACCAGGAAACAGCTATGAAAGACCTACGTTCTCATTTTCTCGAC |
| M1-*cfa*2-R | cctggaatgcaggggagcggcaagaTCAAAGTTCGCCCCAATCGGG |
| Up_500_-F | CAGGATCCGAATTCGAGCaaacctttacgcgtaatgcgtgg |
| Up_500_-R | CTCTTGTCAACACCGCCAGAGATAACTTTTCAAAGAAAAACACCGTTCAATTTGAACGGTGTCCTGagttttagcggttttttccgtcagc |
| Down_500_-F | CCCGATTGGGGCGAACTTtgatcttgccgctcccctgcattc |
| Down_500_-R | AAGCTTGTCGACCTGCAGtgtctgttttgcggtcgccag |
| Linear-*cfa*2-HF-F | aaacctttacgcgtaatgcgtgg |
| Linear-*cfa*2-HF-R | tgtctgttttgcggtcgccag |
| pTarget-N_20_ | CTATGAGAAAGCGCCACGCTTC |
| pTargetF-*ldhA*-N_20_- F | CTGGATCATAGGCTGGAACAGTTTTAGAGCTAGAAATAGCAAGTT |
| pTargetF-*ldhA*-N_20_-R | tgttccagcctatgatccagACTAGTATTATACCTAGGACTGAGC |

^a^Homologous sequences for recombination, overlap sequences for overlap extension PCR and the “N_20_” sequence for the target *ldhA* are underlined (where N_20_ represents the 20bp complementary region and N represents any nucleotide). The start codon (ATG) and stop codon (TCA) of the *cfa*1 and *cfa*2 genes are shown in bold, and the BT5 terminator sequence is shown in red.

**Supplementary Figures**

Supplementary Figure S1. The catalytic mechanism of CfaS. The light blue gradient background shows AdoMet and its catalyzed product, S-adenosyl-L-homocysteine (AdoHcy).

**a**


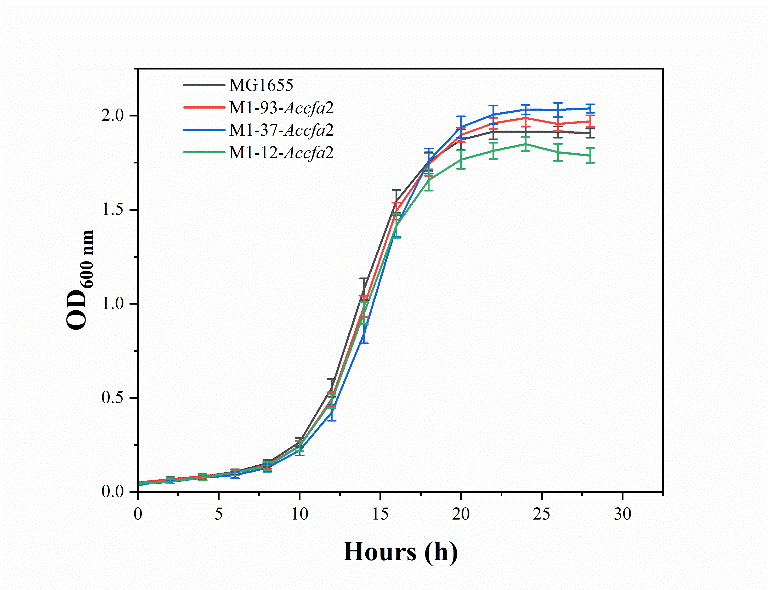


**b**


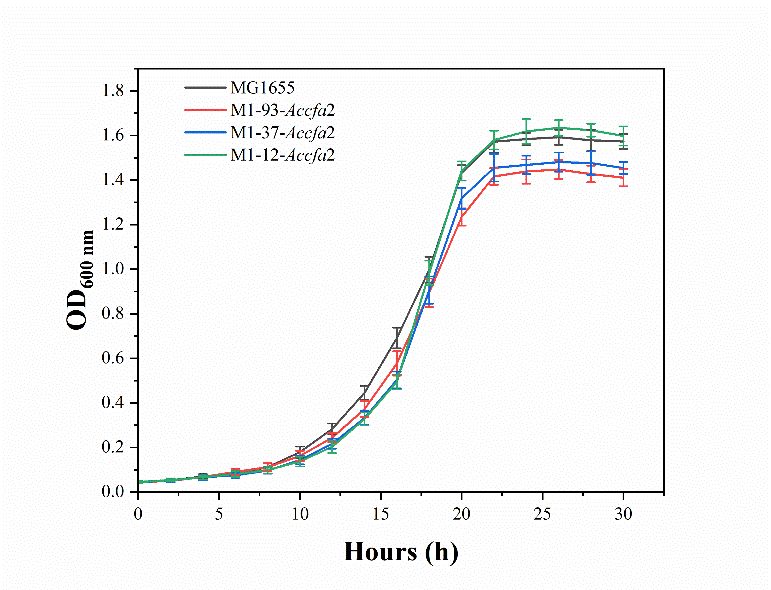


**c**


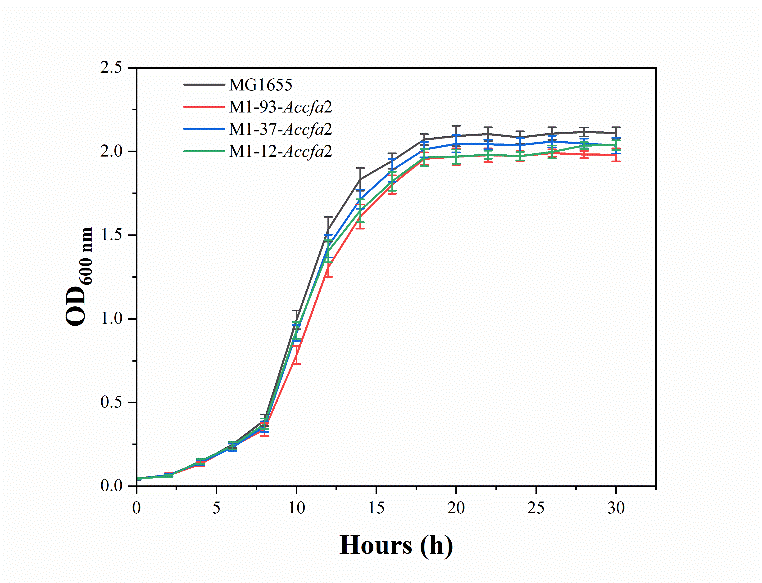


**d**


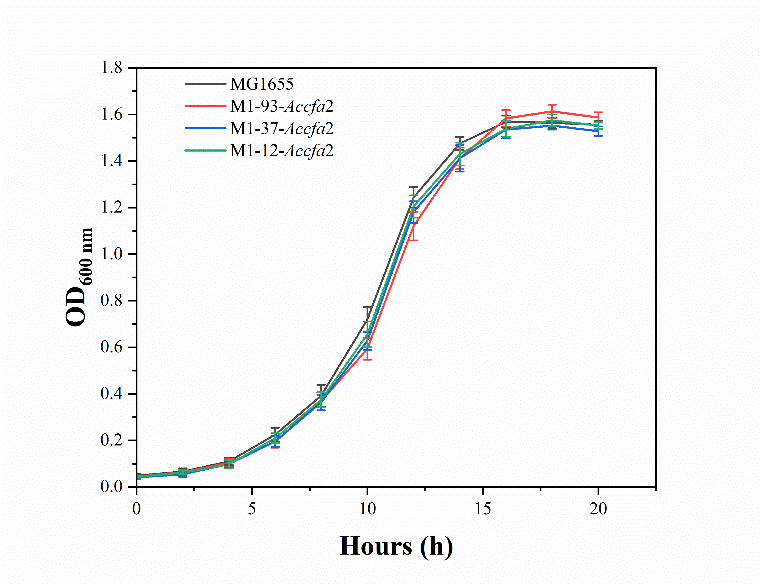


**e**


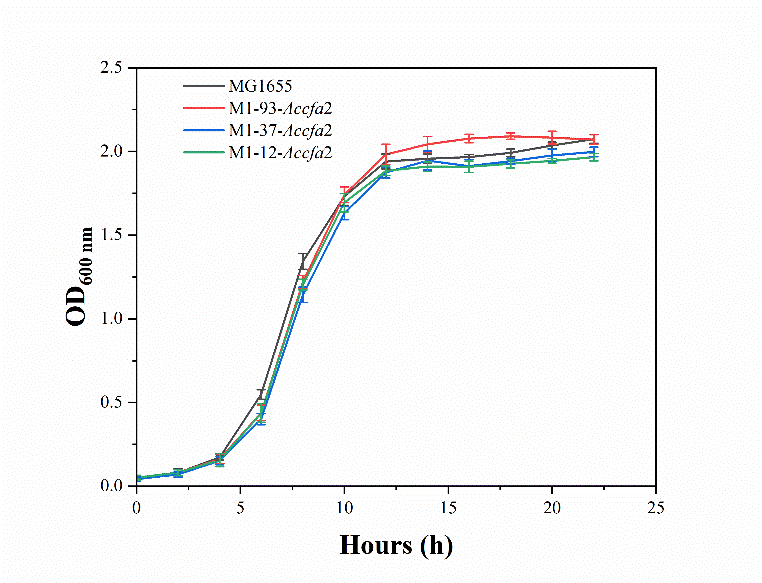


**f**


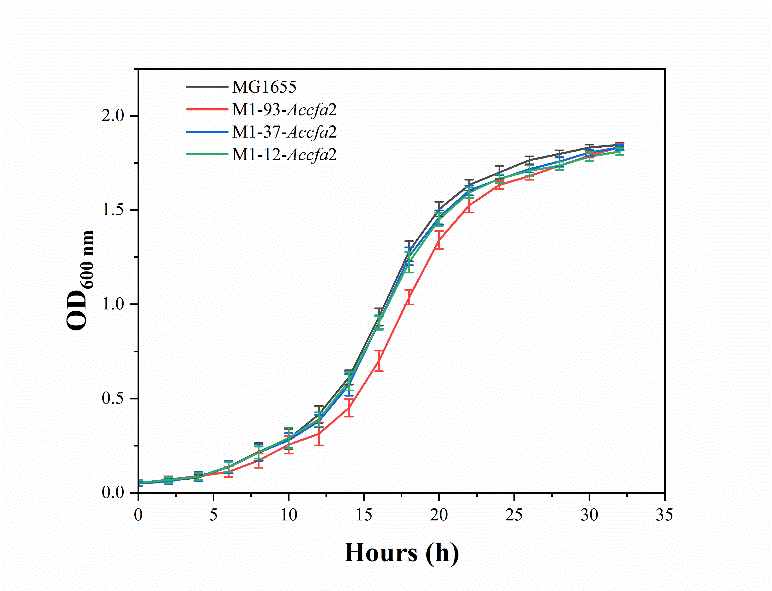


Supplementary Figure S2. A universal study of the role of CfaS-based membrane engineering strategy in improving microbial robustness. Four general classes of inhibitory compounds (**a-d** represent organic acids, short-chain fatty acids, alcohols, and aromatics, respectively) as well as two classes of environmental stressors (**e** and **f** represent high temperature and high osmotic pressure, respectively) were employed. The final concentrations of each chemical are as follows. acetate, 15 mM; octanoic acid, 10 mM; ethanol, 2%(v/v), 343 mM; phenol, 11 mM; sodium chloride, 600 mM. In this study, 42 °C was set as the temperature required for high temperature stress.
